# Optimization of Production Methods for Black Soldier Fly Larvae (*Hermetia illucens* L.) in Burkina Faso

**DOI:** 10.3390/insects14090776

**Published:** 2023-09-21

**Authors:** Florence Sankara, Fernand Sankara, Salimata Pousga, Kalifa Coulibaly, Jacques Philippe Nacoulma, Zakaria Ilboudo, Issoufou Ouédraogo, Irénée Somda, Marc Kenis

**Affiliations:** 1 Institut du Développement Rural, Université Nazi Boni, Bobo-Dioulasso 01 BP 1091, Burkina Faso; florencesankara21@gmail.com (F.S.); ferdisank2005@yahoo.fr (F.S.); pousgasalimata@yahoo.fr (S.P.); coulkal1@gmail.com (K.C.); nacphil2@yahoo.fr (J.P.N.); ireneesomda@yahoo.fr (I.S.); 2 Laboratoire d’Entomologie Fondamentale et Appliquée, Université Joseph Ki-Zerbo, Ouagadougou 06 BP 9499, Burkina Faso; il_zakaria@yahoo.fr; 3 Institut de l’Environnement et de Recherches Agricoles, Bobo-Dioulasso 01 PB 910, Burkina Faso; yeguereo@gmail.com; 4 CABI, 1 Rue des Grillons, 2800 Delémont, Switzerland

**Keywords:** *Hermetia illucens*, proteins, substrates, containers, poultry feed, Burkina Faso

## Abstract

**Simple Summary:**

The black soldier fly is a tropical and subtropical fly that is increasingly used in animal feed worldwide. Its larvae develop in organic plant or animal matter and agro-industrial by-products. In Africa, they represent a source of protein that can help improve local poultry feed rations. The aim of this study was to improve the technique of black soldier fly larvae production by using local substrates (poultry droppings, cotton cake, brewery waste, and local beer waste) for animal feed in Burkina Faso. This study showed that the production of black soldier fly larvae by exposing substrates to naturally occurring flies is possible but that yields strongly vary according to the season and the substrates and types of containers used. These results provide important information for the development of sustainable insect-based poultry feed production methods in Sub-Saharan Africa.

**Abstract:**

Larvae of *Hermetia illucens* are a valuable source of protein for animal feed that can be produced by exposing animal and agro-industrial wastes to naturally occurring flies. The objective of this study was to improve techniques for obtaining *H. illucens* larvae to feed livestock in Burkina Faso. An experiment was conducted to determine the most favourable substrates and seasons for larval production. The substrates used were poultry manure, local beer waste, local beer waste mixed with poultry manure, cottonseed cake, and industrial brewery waste mixed with poultry manure. The production of larvae was carried out in four different seasons. The effect of the container’s oviposition area (0.07 m^2^, 0.09 m^2^, and 0.11 m^2^) and the type of container (terracotta, plastic, and iron) on larval production was also assessed. The produced larval biomass was high during, or just after, the rainy season but very low during the cool dry and hot dry seasons. Yields were higher with local beer waste mixed with poultry manure followed by local beer waste and cottonseed cake. The average mass of *H. illucens* larvae increased slightly with the oviposition area for the same amount of substrate. Iron and terracotta containers provided better results than plastic containers. The suitability of this production method for *H. illucens* larvae production is discussed.

## 1. Introduction

The UN estimates that the world’s population is expected to reach 8.5 billion by 2030 and 9.7 billion by 2050 [1]. As the world population increases, so does the need for human food and animal feed [2]. To meet this growing demand, the development of alternative food and feed production methods is essential. Insects are increasingly seen as a new source of protein for human food and animal feed [3]. Insects, such as termites, housefly (*Musca domestica* L. (Diptera: Muscidae)) larvae, and black soldier fly (*Hermetia illucens* (L.) (Diptera: Stratiomyidae)) larvae are very rich in protein and dietary fat and can be used in animal feed to reduce the use of unsustainable protein sources, such as fishmeal and soybean [4,5]. *Hermetia illucens* larvae are particularly popular because they can be produced on various wastes, such as food waste, livestock manure, and agro-industrial waste [6,7,8,9]. Dry or fresh larvae can be provided as protein sources to monogastric animals, such as poultry, pigs, and fish [10,11,12,13].

Nowadays, *H. illucens* larvae are produced in large quantities in several countries around the world [5,14,15]. Protein production is not the only aim of *H. illucens* production plants. The production residues can be sold as excellent bio-fertilizers, and *H. illucens* can also be used for recycling large amounts of organic wastes [16,17,18]. In contrast to most other flies, adult black soldier flies feed only on water [10], surviving on fat stored during their larval stage, and they are neither pests nor disease vectors. Larvae also have the potential to reduce harmful bacteria and housefly populations [3]. *Hermetia illucens* larvae contain high levels of lauric acid, which has an antimicrobial effect on intestinal pathogens [19]. These larvae are therefore capable of reducing or neutralizing most disease-transmitting bacteria, such as *Salmonella* spp. and *Escherichia coli* [20,21,22]. This limits the risk of disease transmission to animals and humans.

*Hermetia illucens* is present in Burkina Faso but not yet used in the country [23]. Including *H. illucens* larvae, and insects in general, in animal feed in Burkina Faso could help with solving the protein deficiencies in livestock feed and reducing the livestock production costs for farmers. To achieve this, simple, efficient, and cost-effective production methods need to be made available to producers [14]. Two types of methods are used for the production of *H. illucens* larvae [14]. In most production systems, adults are reared in captivity, and the harvested eggs are deposited on a suitable substrate (e.g., [24]). However, small-scale producers can also use an open system and attract adult oviposition in the wild by exposing substrates on which the flies will lay eggs and the larvae will develop [14,25,26]. This open system can be a large, semi-closed container that is regularly refilled with substrates and from which mature larvae can extract themselves (e.g., [25]), but *H. illucens* can also be obtained by farmers simply by exposing substrates in open containers from which the larvae will be extracted with sieves [26,27]. In such open systems, it is important to select the best available substrates and the most efficient types of containers in different climatic environments and seasons. Such information is still largely unknown, especially in West Africa. This study was conducted in an open system to (a) assess the effect of substrates and the period of the year on *H. illucens* larvae production, (b) assess the effect of the oviposition area on *H. illucens* larvae production, and (c) assess the effect of the type of rearing container on the fresh larval biomass of *H. illucens*.

## 2. Materials and Methods

### 2.1. Experimentation Area

**Figure 1:**
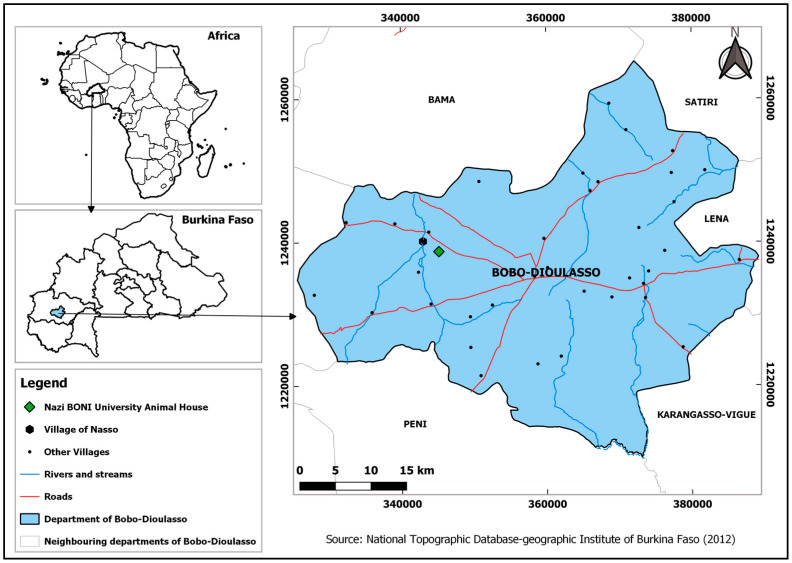
Representative map of the experimental area.

This study was carried out at the site of the Nazi BONI University (UNB) animal house located in the village of Nasso (11°12′ N, 4°25′ W). Belonging to the commune of Bobo-Dioulasso and the Hauts-Bassins region, this village is located about fifteen kilometres from the city (Figure 1). This area is characterized by a south Sudanese climate and by a dry season (October to April) and a rainy season (May to September). The vegetation consists of wooded savannahs, trees, and shrubs [28].

### 2.2. Influence of Substrates and Seasons on the Production of H. illucens Larvae

**Figure 2:**
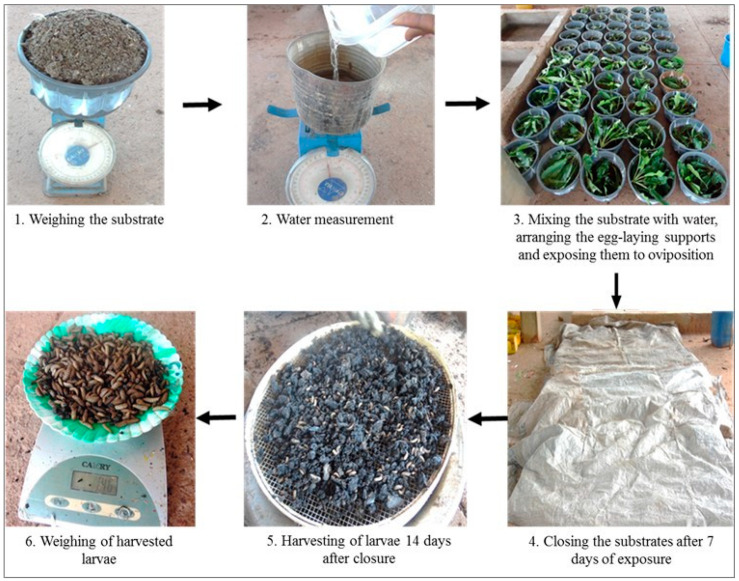
Production process of black soldier fly larvae.

To study the influence of substrates and seasons of the year on *H. illucens* larvae production, 100 *H. illucens* larvae production containers were placed in the open air and protected from the rain by a roof. These containers were arranged in a completely randomized block design with five treatments and five replicates per treatment. Four successive replicates were conducted. The treatments consisted of the following substrates: poultry manure, local beer waste, local beer waste mixed with poultry manure, cottonseed cake, and industrial brewery waste mixed with poultry manure. The different substrates were placed in plastic containers (diameter: 27.5 cm; depth: 20 cm) and replicated 20 times. Two kg of each substrate was mixed with water (3 L), except in the local beer waste, which was already damp, where 4 kg was weighed and mixed with 1 L of water. Four cardboard pieces (approximately 8 × 4 cm) and a cluster of 10 to 15 shea tree leaves (*Vitelaria paradoxa*) were placed on the surface of the substrates to attract adult flies for oviposition. Seven days after exposure, the containers were covered with ventilated plastic sheets made of old cereal bags. The larvae were harvested 14 days after having covered the substrates. Sieves with a mesh size of 0.4 to 0.6 cm were used to collect the larvae following the method described by [29] and [30]. This experiment was repeated in July–August 2020, October–November 2020, January–February 2021, and April–May 2021, (i.e., four times in the years 2020 and 2021). The production process of black soldier fly larvae is summarized in Figure 2. The parameter measured was the fresh larval biomass harvested according to the substrates and the period. The temperature and air humidity in the production area were recorded each morning between 8 and 9 a.m. and each afternoon between 2 and 3 p.m.

### 2.3. Influence of the Oviposition Surface on the Production of H. illucens Larvae

**Figure 3:**
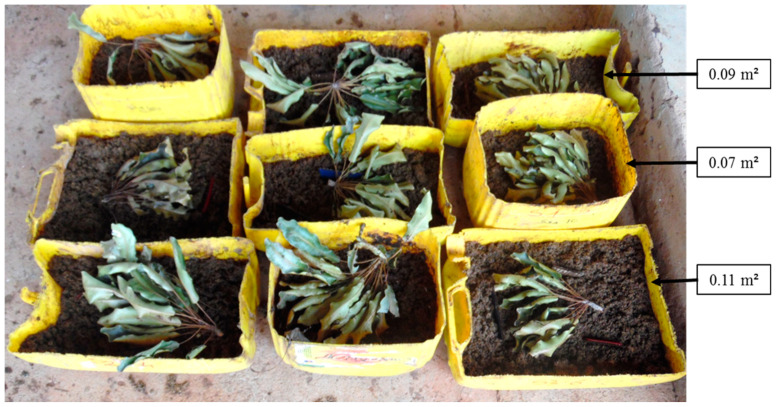
Device for testing the influence of the oviposition surface on the production of *H. illucens* larvae.

To evaluate the effect of the oviposition surface on *H. illucens* larval production, 20 L plastic jerrycans were cut to obtain containers with three different surfaces: 0.07 m^2^ (≈0.2 m deep); 0.09 m^2^ (≈0.15 m deep); and 0.11 m^2^ (≈0.10 m deep). Local beer waste mixed with poultry manure was used as a substrate for the production of *H. illucens* larvae. In total, 2 kg of wet local beer waste was mixed with 1 kg of poultry manure and 1.5 L of water and put into each container. In addition, four cardboard pieces (approximately 8 × 4 cm) and a cluster of 10 to 15 shea tree leaves were placed on the surface of each substrate to attract adult flies for oviposition. This test was carried out in a completely randomized design with three treatments (oviposition area), and each treatment was repeated three times (Figure 3). Four successive replicates were conducted. After seven days of exposure, the substrates were covered with ventilated plastic sheets made of old cereal bags. Larvae were harvested with sieves 14 days after having covered the substrates. This experiment was carried out in July 2021, September 2021, and November 2021. The parameters measured were the fresh larval biomass and the weight of 100 larvae. The number of larvae obtained per exposure area was estimated according to the following formula: Number of larvae = (fresh larval biomass × 100)/weight of 100 larvae.

### 2.4. Influence of the Nature of the Container on the Production of H. illucens Larvae

**Figure 4:**
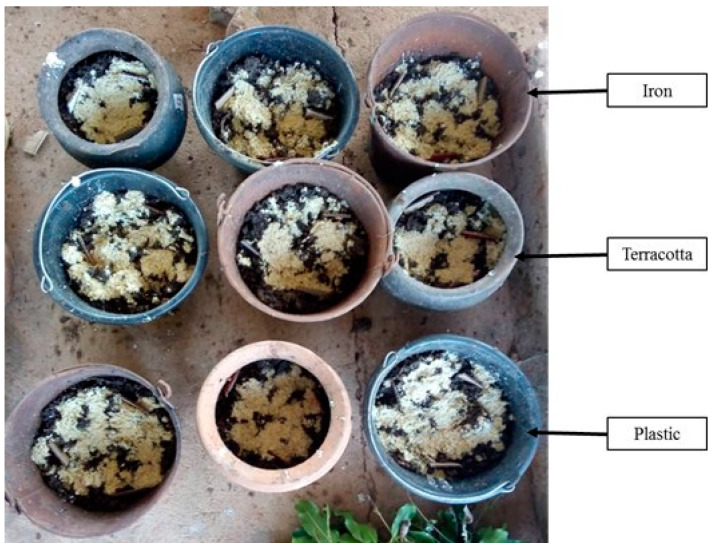
Device for testing the influence of the nature of the container on the production of *H. illucens* larvae.

To evaluate the effect of the type of container on the production of *H. illucens* larvae, terracotta (canary; diameter: 25.5 cm; depth: 26 cm), plastic (bucket; diameter: 27.5 cm; depth: 22 cm) and iron (bucket; diameter: 28 cm; depth: 24.5 cm) containers were used. In this test, the substrate for the production of *H. illucens* larvae was local beer waste (2 kg) mixed with poultry manure (1 kg). Also, four cardboard pieces (approximately 8 × 4 cm) and a cluster of 10 to 15 shea tree leaves were placed on the surface of each container to attract adult flies for oviposition. The experimental design consisted of completely randomized blocks with 3 treatments (type of container) and 3 replicates per treatment [29] (Figure 4). Four successive replicates were conducted. As for the other experiments, the containers were covered after 7 days, and larvae were harvested 14 days later using sieves. This experiment was carried out in July 2021, September 2021, and November 2021, and the parameters measured were fresh larval biomass, the weight of 100 larvae, and the number of larvae obtained per type of container.

### 2.5. Data Analysis

R software version 4.2.1 [31] was used for data analysis. The Shapiro–Wilk and Bartlett tests were performed to check the normality of the data and the homogeneity of the variances, respectively. In cases where the data followed the normal distribution, to assess the different factors (substrates, production period, oviposition area, and containers), a one-way ANOVA (Analysis of Variances) test was used at the 5% probability level followed by Tukey’s HSD post hoc test for the separation of means when significant differences were observed. In cases where the data did not follow the normal distribution, the Kruskal–Wallis test was used to separate the means at the 5% probability level.

## 3. Results

### 3.1. Influence of Substrates and Period on the Production of H. illucens Larvae

**Figure 5:**
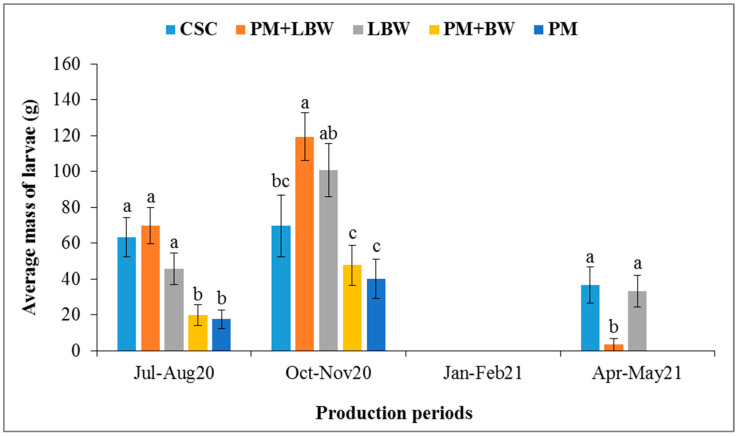
Average mass of harvested larvae (±standard error) as a function of production substrates by period. CSC = cottonseed cake; PM = poultry manure; LBW = local beer waste; BW = brewery waste. For each production period, means surmounted by the same letter are not significantly different at the 0.05 level.

**Figure 6:**
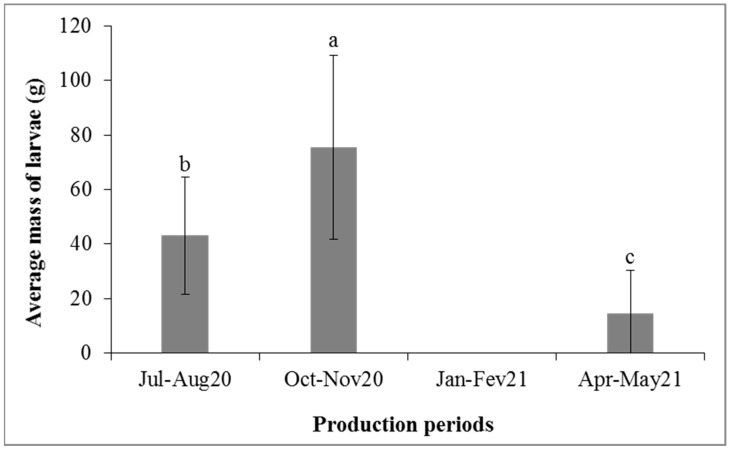
Average mass of harvested larvae (±standard error) as a function of production periods. The means surmounted by the same letter are not significantly different at the 0.05 level.

Figure 5 and Figure 6 show the average masses of the *H. illucens* larvae harvested as a function of the production substrates by period and the average masses of the *H. illucens* larvae harvested as a function of the production periods, respectively. Significant differences were found between production substrates (*p* < 0.0001) and between production periods (*p* < 0.0001). The most productive periods in terms of *H. illucens* larvae were the months of October–November 2020 with certain substrates, such as poultry manure mixed with local beer waste (119.4 g of larvae), local beer waste (100.7 g), and cottonseed cake (69.6 g), followed by the period of July–August 2020, with the same substrates producing 69.6 g, 45.7 g, and 63.3 g, respectively. *Hermetia illucens* larvae production was low in April–May 2021, and no larvae were obtained in January–February 2021.

**Figure 7:**
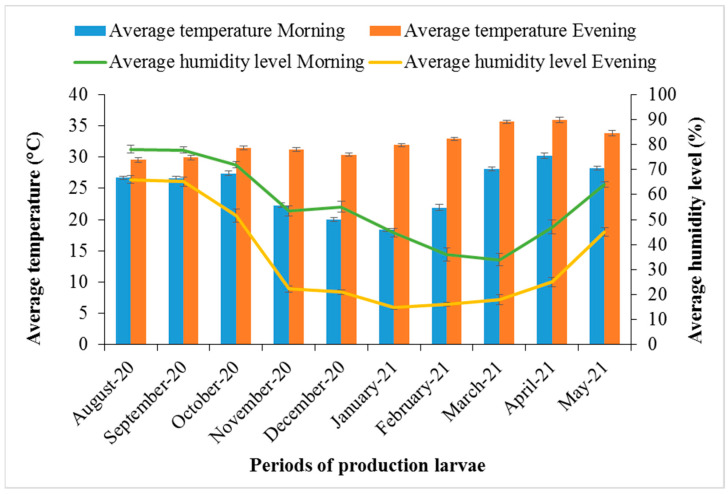
Average temperatures and humidity levels recorded during the production of *H. illucens* larvae from August 2020 to May 2021.

Figure 7 shows the monthly average temperatures and humidity levels recorded in the mornings and evenings during the production of *H. illucens* larvae. The highest temperature was recorded in April in both the morning (30.2 °C) and the evening (35.9 °C), and the lowest was recorded in January (18.6 °C) in the morning and in July and August in the evening (29.6 °C). The highest humidity was recorded in August (78% in the morning and 65.9% in the evening) and the lowest was recorded in February (26%) in the morning and in January and February (14.8%) in the evening.

### 3.2. Influence of the Oviposition Area on the Production of H. illucens Larvae

**Figure 8:**
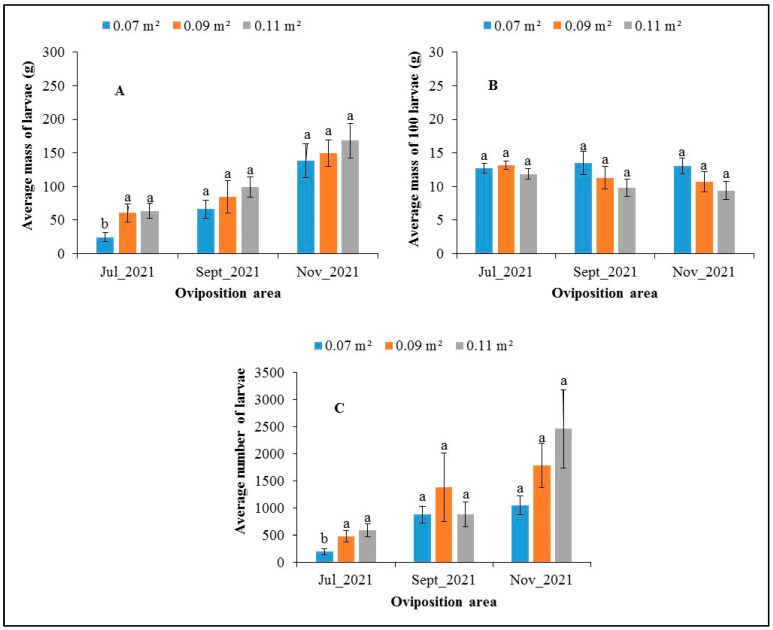
Average mass of larvae (**A**), mass of 100 larvae (**B**), and number of larvae (**C**) (±standard error) of *H. illucens* as a function of the oviposition area at three different months. For each period, the means surmounted by the same letter are not significantly different at the 5% level.

The histograms in Figure 8 show the fresh larval biomass (A), the average mass of one hundred larvae (B), and the average number of *H. illucens* larvae (C) produced as a function of the oviposition area and the production period. The average mass and average number of *H. illucens* larvae increased from July to November (Figure 8A,C). In July 2021, the Analysis of Variance showed a statistically significant difference (*p* = 0.019) between the 0.07 m^2^ area (24.3 g) and the other two areas, with 61 g for 0.09 m^2^ and 63.9 g for 0.11 m^2^. This was also the case for the average number of larvae produced. For the months of September and November 2021, no significant difference was found between the different areas in terms of the average masses of larvae produced and the average number of larvae produced. However, the trends showed that the largest area, 0.11 m^2^, produced the highest number of larvae in terms of quantity and number, except in the month of September. As for the average mass of one hundred larvae, the statistical analysis showed no significant differences (*p* ˃ 0.1) between the production months or between the oviposition areas. The average mass of the one hundred larvae hovered around 12.5 g (Figure 8B). The trends showed that the larger the oviposition area, the lower the mass of one hundred larvae.

### 3.3. Influence of the Type of Container on the Production of H. illucens Larvae

**Figure 9:**
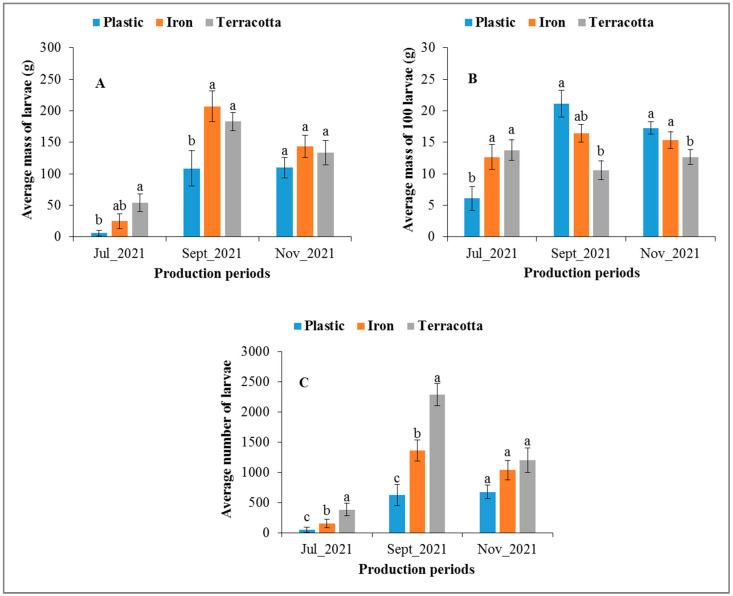
Average mass of larvae (**A**), mass of 100 larvae (**B**), and number of larvae (**C**) (± standard error) of *H. illucens* as a function of the type of container at three different months. For each period, the means surmounted by the same letter are not significantly different at the 5% level.

The histograms in Figure 9 show the fresh larval biomass (A), the average mass of one hundred larvae (B), and the average number of *H. illucens* larvae (C) produced according to the nature of the container and the period of production. The average mass of *H. illucens* larvae, the mass of one hundred larvae, and the average number of larvae were lower in July than in September and November (Figure 9A–C). The average mass of the larvae obtained differed significantly between the production containers in July (*p* = 0.0033) and September (*p* < 0.0001) but not in November (*p* = 0.403). The larval biomass produced was lower in plastic containers (5.8 g, 108.6 g, and 109.8 g, respectively, for the months of July, September, and November) than in iron (24.6 g, 207.1 g, and 143.6 g) and terracotta (54.1 g, 182.8 g, and 133.8 g) containers (Figure 9A). The mass of one hundred larvae was higher in the plastic containers (21.1 g and 17.3 g), except in the month of July (6.1 g) (Figure 9B). The number of larvae was higher in terracotta containers (382.3 g, 2285.4 and 1197.2 larvae) (Figure 9C).

## 4. Discussion

This study showed that the production of *H. illucens* larvae in a semi-natural environment based on wild females varies with the substrate used, the season, the oviposition surface, and the type of container used for oviposition. As expected, the different substrates used for producing *H. illucens* larvae provided different quantities of larvae. This was also observed in many other studies (e.g., [6,25,26,32]). However, it is not clear whether these differences are due mostly to the differences in attractiveness to the females and, thus, the quantity of eggs laid, or due to the quality of the substrate itself for larval development. Boafo et al. [26] recently found that the most attractive substrates for *H. illucens* are not necessarily the best for larval development. Local beer waste mixed with poultry manure, local beer waste alone, and cottonseed cake produced a higher quantity of larvae than the other substrates (poultry manure and industrial brewery waste mixed with poultry manure). This may be because these substrates produce a very strong fermentation smell, which would attract flies to lay eggs. In addition, these substrates are very rich in nutrients and are not too compact, which favours larval development. Our results are in accordance with those of [25,33,34,35], who reported that *H. illucens* larvae develop less well on substrates of animal origin than on those of plant origin.

The lower performance of substrates based on poultry manure in our experiments may also be because poultry manure is a preferred substrate for house flies, *M. domestica* [36]. Indeed, when substrates are exposed in open systems, such as the ones used in our study, *M. domestica* is the first insect to colonize the substrates, and its larvae develop very rapidly, within three or four days. *Hermetia illucens* eggs take about four days to hatch, and larvae are usually observed after a week, when *M. domestica* has already pupated [27]. Thus, *M. domestica* larvae may have already partly exhausted the substrates before the development of *H. illucens* larvae. In general, open systems for *H. illucens* production involve containers that are regularly refilled with substrates and from which *H. illucens* larvae exit by themselves [25,26]. In such systems, *H. illucens* is highly competitive and can prevent the occurrence of *M. domestica* larvae, resulting in a higher production of *H. illucens* larvae [37]. In competitive interaction experiments conducted in poultry manure, Miranda et al. [37] showed that the development and survival of *M. domestica* did not significantly differ when it colonized the poultry manure first or within two days of the initial introduction of *H. illucens*. Conversely, the development and survival of *H. illucens* were negatively affected when *M. domestica* colonized the substrate first or within four days of the introduction of *H. illucens*. The poor performance of *H. illucens* in the latter case is likely the result of reduced moisture and nutrient content in the poultry manure due to *M. domestica* larval activity.

Our results also show that the productivity of *H. illucens* larvae strongly varies with the season. Indeed, the productivity of the larvae was highest in the October–November period, followed by the July–August period (i.e., during or just after the rainy season, when the relative humidity is still high). No larvae were produced in the cool dry season in January–February, and only few larvae were produced during the hot dry season in April–May. This is likely because of the impact of the temperature and humidity on *H. illucens* adults and larvae. Indeed, Tomberlin et al. [34] and Sheppard et al. [38] have shown that *H. illucens* larvae develop optimally at temperatures between 27 and 30 °C and humidity between 60 and 90%. Bullock et al. [39] also found that oviposition periods depend on relative humidity and temperature. *Hermetia illucens* is naturally found only in tropical, sub-tropical, and warm temperate climates, and it does not like very dry climates [16], even though it can survive cold temperatures by slowing down its development rate, without reproducing [39]. Park et al. [40] also found that the reproductive behaviour of *H. illucens* is determined by seasonal variations and, more specifically, by the decrease in day length and light intensity. However, in Burkina Faso, a tropical country, rainfall and relative humidity are probably the most important climatic factors, because the fly is apparently rare in the long dry season. In southern Ghana, where the relative humidity is high throughout the year and the dry seasons are short, *H. illucens* is found ovipositing during the whole year [41].

The absence or low frequency of *H. illucens* oviposition during most of the dry season in Burkina Faso suggests that it may be difficult to develop a sustainable *H. illucens* production system based on naturally occurring flies in the region. Rather, *H. illucens* should be produced during the whole year with adults reared in captivity in favourable conditions of humidity and temperature. Such production systems exist everywhere in the world and at various scales. However, the most efficient and cost-effective techniques still need to be developed for farmers and entrepreneurs in Burkina Faso while considering the availability and costs of efficient substrates, the costs of temperature and humidity control, and various other factors. For West African farmers, even the smallest rearing system in captivity is costly and time consuming. Those who prefer simple production systems may consider producing house flies. In similar climatic conditions in Bamako (Mali), Koné et al. [36] succeeded in obtaining high amounts of *M. domestica* larvae throughout the year by exposing substrates to naturally occurring house flies.

Larval production only weakly increased with the oviposition area, all other factors being equal. This can be explained by the fact that females do not lay eggs directly on the substrate, but rather on drier oviposition supports, such as cracks and crevices in the vicinity of decaying organic matter [42], and our oviposition structures placed in the containers did not vary with the oviposition area. A similar experiment with *M. domestica* [29] showed a stronger positive reaction to increasing the oviposition area, probably because *M. domestica* directly oviposits on the substrate.

The production of *H. illucens* larvae varied according to the nature of the containers used. The average mass and number of *H. illucens* larvae in the iron and terracotta containers were higher than in the plastic containers. This can be explained by the fact that the different containers regulate the temperature and humidity differently. The iron container lets heat escape, the terracotta container retains moisture, and the plastic container retains heat. Dortmans et al. [24] suggest that the moisture content of diets for *H. illucens* larvae should generally be between 70 and 80%. According to Kenis et al. [16], for rearing *H. illucens* larvae, plastic containers are not desirable because the larval activity strongly heats up the environment and *H. illucens* larvae are sensitive to high temperatures. Therefore, iron containers, which allow heat to escape, are preferred to plastic containers. The weight of one hundred larvae in the plastic containers was higher compared to the iron and terracotta containers, probably because less larvae survived in the plastic containers, resulting in reduced intra-specific competition. In another study, Baragan et al. [43] showed that, at high densities, competition for nutrients can negatively influence the growth of *H. illucens* larvae. Intra-specific competition is also likely the reason why, in several of our experiments, when the average mass of all larvae was high, the mass of 100 larvae was comparatively low. Thus, for specific rearing conditions (substrate, season, and container), an ideal amount of substrate should be calculated to minimize the effect of intra-specific competition. Or, a method can be developed that would add specific amounts of substrates in the system, as is performed in semi-closed containers, such as those in [25].

## 5. Conclusions

The production of *H. illucens* larvae can be simultaneously used for waste management, for the production of animal protein, and for the production of biofertilizers, making it a highly beneficial insect. This study showed that the simple exposure of substrates to natural oviposition can produce black soldier fly larvae. However, production highly depends on the substrate used, the type of container, and the season. In Burkina Faso, the low oviposition rate of naturally occurring females during the dry season impedes the use of production methods based on natural oviposition. Instead, simple larvae production methods already used elsewhere, based on eggs produced by females in captivity, should be adapted for the specific climatic conditions and farming systems in Burkina Faso.

## Data Availability

The datasets analyzed during the present study are available upon request from the first and second authors.
